# Investigating Fast Scanning Calorimetry and Differential Scanning Calorimetry as Screening Tools for Thermoset Polymer Material Compatibility with Laser-Based Powder Bed Fusion

**DOI:** 10.1021/acsapm.4c03052

**Published:** 2025-01-13

**Authors:** Malik A. Blackman, Meisha L. Shofner, Camden A. Chatham

**Affiliations:** † School of Materials Science and Engineering, Georgia Institute of Technology, Atlanta, Georgia 30332, United States; ‡ Advanced Engineering Division, Savannah River National Laboratory, Savannah River Site, Aiken, South Carolina 29808, United States

**Keywords:** fast scanning calorimetry, differential scanning calorimetry, thermoset, curing kinetics, isoconversional analysis, powder bed fusion, additive manufacturing

## Abstract

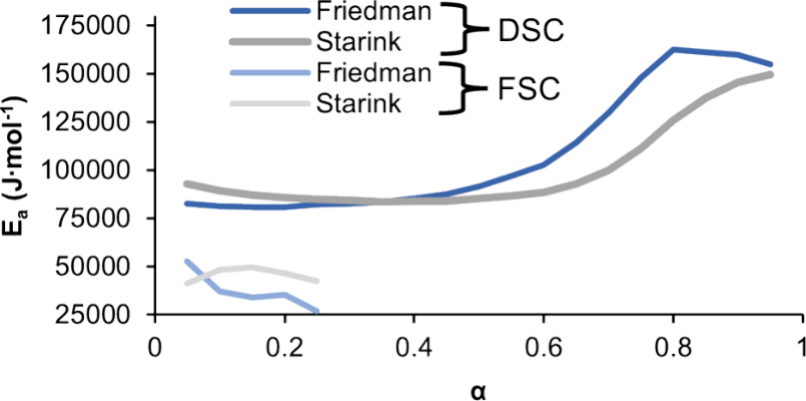

As additive manufacturing (AM) technology has developed and progressed, a constant topic of research in the area is expanding the library of materials to be used with these techniques. Among AM methods that utilize polymers, laser-based powder bed fusion (PBF-LB) has preferentially used thermoplastic polymers as its starting materials, but the deposition and material joining method employed in PBF-LB may also be compatible with powdered thermoset polymer precursors as feedstocks. To assess the compatibility of candidate thermosetting polymers and PBF-LB, characterization techniques and protocols that link fundamental material behavior to material behavior in the processing environment are needed. Therefore, the objectives of this work are to compare the curing behavior measured with two different calorimetry techniques that can operate in different heating rate regimes, differential scanning calorimetry (DSC) and fast scanning calorimetry (FSC), and to assess the capabilities of these techniques to act as materials screening tools for PBF-LB. A commercial polyester powder coating is used as a model material to evaluate the potential of obtaining complementary information for material screening through a combination of calorimetry methods, and its nonisothermal curing behavior is measured at heating rates between 5 and 7500 °C/min. Curing exotherms are observed with both calorimetry techniques, and comparing the enthalpy associated with curing shows that incomplete curing occurs at higher heating rates, with relative conversion values of approximately 30%. The curing data are fit with two isoconversional models, Friedman and Starink, which show a reduced activation energy at higher heating rates as well, signifying a lower barrier to curing at the conditions used in the FSC experiments. Overall, the results of this work indicate that using these two calorimetry techniques as tiered screening tools can provide valuable information about how curing may proceed in PBF-LB and inform materials selection and design activities for additive manufacturing.

## 1 Introduction

Additive manufacturing (AM) technologies have evolved from their beginnings as prototyping tools to methods for end-use manufacturing of parts in an ever-growing number of industries, including biomedical,^1,2^ textiles,^3,4^ and aerospace.^5,6^ AM has been championed for its ability to manufacture increasingly complex parts without the need for complex molds or tooling. Such potential has translated into the need for increased development of high-performance polymeric materials in AM. However, the expanded use of polymer-based AM has required greater consideration of material microstructure as it pertains to improving part production, meaning that the evolution of material structure and properties with respect to fabrication method parameters must be well understood.^7,8^

The AM method of interest for this work is laser-based powder bed fusion (PBF-LB). PBF-LB is also known by its legacy term Selective Laser Sintering or SLS.^9^ As the name suggests, a laser is utilized to selectively heat and fuse powdered material to form a three-dimensional part. The process proceeds like other AM methods by constructing the part in layers, with this method using the laser to fuse the powdered material within a single layer and between adjacent layers. PBF-LB has often been advocated for due to its large build volume and low anisotropy when compared to other AM processes during end-part production.^10,11^ Additionally, the use of a powder feedstock eliminates the need for support structures, and powders are easily mixable, allowing for end-part printing using more than one material.^12^ While thermoplastics currently dominate the polymeric feedstock choices for PBF-LB, with commercially available options such as polyamides and polypropylene,^13,14^ other polymer feedstock options are of interest to increase the addressable application space for PBF-LB parts. In harsh environments, materials with greater chemical resistance, higher service temperatures, and higher modulus would be required, which are properties typically associated with thermosets. Unlike thermoplastics that transition from a solid to a liquid as temperature is increased, the covalently bonded network of polymer chains in a thermoset cannot be thermally deconstructed into mobile polymer chains. Limited research has been conducted with thermoset feedstocks for PBF-LB, and these studies have identified challenges specific to thermoset feedstock materials and shown promising results. Research with a high temperature imide thermoset, Resin Transfer Molding (RTM) 370 Resin, illustrated how a material like this could be integrated into PBF-LB, specifically by including thermal staging steps before laser scanning to increase viscosity and incorporating carbon fiber to improve thermal transport during laser scanning.^15,16^ Ultimately, the researchers were able to successfully print a composite part and apply postcuring steps to produce a dense part with a thermosetting resin. Additionally, PBF-LB processing studies have been conducted with a bismaleimide resin powder.^17^ This work also identified strategies for processing this material that included the addition of a carbon-based filler and postcuring. Another strategy that was investigated to use thermosets with PBF-LB involved precuring an epoxy resin/curing agent mixture containing a small amount of carbon black to a conversion level of ∼50%, powdering the precured mixture, and then sintering the powder with PBF-LB.^18^ In this approach, the PBF-LB process was not used to cure the material further, only consolidate and fuse the powdered particles, and full curing was achieved in through a postprocessing heating step. Recognizing the increasing interest in thermosets for PBF-LB, companies have begun marketing thermoset feedstocks, highlighting further opportunities for part production with this AM method.

In addition to studies focused on individual feedstock materials of interest, it is important to examine the curing behavior of thermosets in PBF-LB from a fundamental perspective that maps the evolution of curing to process steps for PBF-LB. Chatham and Washington^7,19^ explored the compatibility of thermosets with PBF-LB by analyzing the curing behavior of several model thermoset materials. The analysis included printing experiments with in situ temperature measurements and thermal analysis experiments. From the printing experiments, beam speeds were varied, resulting in different temperature profiles during printing which would be expected to affect the degree of cure achieved for each material. Accompanying thermal analysis experiments were performed to obtain the nonisothermal curing behavior and model the curing kinetics of the polymers. The results of this work showed incomplete curing during PBF-LB printing for all of the conditions and for all the thermoset materials investigated, similar to the other experimental efforts described above. This result was an important insight since it indicated that postprocessing heat treatment of parts may be a general need to achieve complete curing. Postprocessing, with light and/or heat, is often required to achieve full cure for thermosetting photopolymers used with vat photopolymerization as well, so having an additional step is not unusual for additive manufacturing of thermosets.^20^ Additionally, modeling of the curing kinetics for the thermosets chosen showed different trends in activation energy as a function of degree of conversion. From the trends shown, it was suggested that thermosets with activation energies that are initially high and then decrease as the curing proceeds may be well suited for PBF-LB processing.^7^ While this research used differential scanning calorimetry (DSC) to examine the curing behavior, which commonly uses heating rates on the order of degrees Celsius per minute, the heating rates associated with laser speeds in PBF-LB are much higher, between 10^3–4^ degrees Celsius per second.^21−23^ To attain a more similar heating environment to PBF-LB and simulate aspects of PBF-LB processing more closely, characterization techniques capable of higher heating rates should be used also. One such characterization technique is fast scanning calorimetry (FSC). FSC can attain heating rates in the order of 100s to 1000s degrees Celsius per second. Published results with FSC in the context of PBF-LB have successfully elucidated the effects of rapid melting and subsequent cooling on the phase behavior of thermoplastic polymers^24,25^ and metals such as aluminum alloys.^26−28^ Combining the results from this method with the results from conventional DSC provides an opportunity to understand if changes in the curing behavior other than lower degrees of conversion would be expected at higher heating rates relevant to PBF-LB.

To address this gap, this study compares the curing behavior of a model thermoset material in DSC and FSC with an aim of relating these results to PBF-LB processing and further understanding the attributes that make thermoset materials viable as PBF-LB feedstocks. Nonisothermal curing experiments were performed at heating rates normally used for thermal analysis with DSC and at higher heating rates with FSC. The curing behavior was analyzed and fit with two isoconversional models to allow comparison between the curing at these different heating rates. The results of the analysis and modeling showed that the material did not cure completely at the higher heating rates and that the activation energy values for curing at higher heating rates were lower than those for conventional heating rates, suggesting some differences in the curing process that could be advantageous for PBF-LB processing.

## 2 Materials and Methods

### 2.1 Materials

Due to the limited availability of thermoset feedstocks for PBF-LB, a commercial powder coating paint was used as the model material in this study. This thermoset material is a triglycidyl isocyanurate (TGIC)-free polyester powder coating purchased from TIGER Drylac (series 61-61/80079). As opposed to TGIC being the cross-linker, β–hydroxyalkylamide (HAA) was the curing agent, with cross-linking reactions expected to start as low as 150 °C. Since uniform particle morphology is important for processing with PBF-LB, particle shape and size were characterized by static image analysis using a Malvern Morphologi 4 instrument. Using the volume distribution of the data, values for D_10_, D_50_, D_90_, and D[4,3] (the volume moment mean diameter) were reported for the powder. Individual particles with circularity <0.66 were excluded from the data set after a manual inspection revealed them to be either artifacts of the image analysis or else contamination with fibers from the cleaning wipes. Additionally, the thermal degradation pattern for the material was characterized by thermogravimetric analysis (TGA) using an SDT Q600 instrument from TA Instruments. In this experiment, the material was heated from 30 to 650 °C at a rate of 10 °C/min. The experiment was performed in a flowing nitrogen atmosphere with a gas flow rate of 100 mL/min.

### 2.2 Calorimetry Experiments

To investigate the curing behavior and provide data for the kinetic analysis, two types of calorimetry experiments were performed: DSC and FSC. DSC experiments were performed using a Mettler Toledo DSC 3+, and FSC experiments were performed using a Mettler Toledo Flash DSC 1. The heating rates used for experiments in this work are shown in Table 1. The DSC data and some kinetic analysis were published previously^7,19^; however, this paper contains a modified analysis of those data for comparison with the FSC data. Specifically, for a subset of the original data set, a different method for isolating the curing peak was used, and different isoconversional models were applied. For each type of calorimetry experiment, three heating rates were used to allow isoconversional kinetic models to be applied to the data. While DSC experiments were conducted at rates typically used for thermal analysis experiments, FSC experiments were conducted at faster rates, meant to approach those used in PBF-LB processing. For DSC, three experiments were performed at each heating rate. These results were averaged for each heating rate and used for further analysis. Results from a single FSC experiment at each heating rate were used for analysis in this work.

**Table 1:** Heating Rates Used in DSC and FSC for Experimental Analysis and Kinetic Modeling

heating rate	DSC	FSC
1	5 °C/min (0.083 °C/s)	50 °C/s (3000 °C/min)
2	10 °C/min (0.17 °C/s)	100 °C/s (6000 °C/min)
3	20 °C/min (0.33 °C/s)	125 °C/s (7500 °C/min)

DSC experiments were conducted at the heating rates shown in Table 1 over temperature ranges specific to each heating rate, detailed in Table S1. For these experiments, samples were placed in aluminum pans and sealed. Sample masses are also given in Table S1. The area under the exothermic curing peak was obtained to determine the energy as a function of time, and these data were used with kinetic models. Thermal exposure from the first heating cycle was assumed to be sufficient to fully cure the material, and heat flow data from a subsequent heating cycle did not show any observable curing, shown in Figure S1.

Since chip sensors were used for the FSC experiments as opposed to sealed sample pans like DSC, it was necessary to perform conditioning steps and a temperature calibration step prior to placing the sample on the chip sensor. The model of the chip sensors used was UFS1. After these steps were complete, the powder was placed on the chip sensor using an eyelash tool, and the sample was heated from 25 to 85 °C at a rate of 1 °C/s, followed by an isothermal hold at 85 °C for 2 s to soften the powder and allow it to adhere to the chip. Then, the sample was heated from 85 to 300 °C at the rates given in Table 1 to cure the material. The exothermic peak associated with curing during this step was isolated and used for kinetic analysis. After the curing step, the sample was cooled from 300 to 40 °C at a rate of 400 °C/s and held at 40 °C for 2 s. Unlike DSC, a subsequent heating cycle was performed to fully cure the sample. In this step, the sample was heated from 40 to 200 °C at a rate of 100 °C/s and held at 200 °C for 80 s to ensure full cure has been reached. The temperature used in this step corresponded to the temperature associated with the fastest curing rate on the product data sheet from the manufacturer. Following this step, the sample was cooled from 200 to 40 °C at a rate of 400 °C/s.

It was necessary to fully cure the material to estimate the mass of the sample since it was not possible to measure the mass of the particles used for the FSC measurements. The method used to estimate mass used the change in the heat flow signal at the glass transition temperature (*T*_g_). The change in the heat flow signal at *T*_g_ of the fully cured FSC samples was compared to the change in the heat flow signal at *T*_g_ of samples fully cured in the DSC with a known sample mass. To obtain the postcuring *T*_g_ with FSC, the sample was heated again from 40 to 300 °C at a rate of 100 °C/s. The post cure *T*_g_ was taken from this heating cycle. The same heating rate was used in this step for all samples. For some samples, these steps were completed within a single experiment, and for others, these steps were accomplished with separate, sequential experiments. The mass calculation was performed using eq 1:
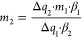
1Where quantities labeled with 1 were obtained from a DSC experiment and those labeled with 2 were obtained from the FSC experiments. In this equation, β is the heating rate, *m* is the mass, and Δ*q* is the heat flow step height value at *T*_g_. The step height in DSC was found by conducting a cyclic heating experiment in a Discovery DSC instrument from TA Instruments, where a heat–cool-heat protocol was used. The powder was first heated from 40 to 300 °C at 50 °C/min to cure the material and then cooled from 300 to 40 °C at 50 °C/min. Finally, the material was heated at a rate of 50 °C/min from 40 to 300 °C, and the *T*_g_ data from this heating step was used in the mass calculation. The mass values obtained from this analysis, shown in Table S2, were then used to normalize the heat flow values obtained by the FSC experiments.

### 2.3 Isoconversional Analysis

Isoconversional analysis is particularly relevant for thermosetting materials as the chemical reactions can be evaluated under the assumption that the reaction model stays consistent throughout the reaction, so the quality of the model fit gives insight into the mechanism of the curing process.^29−31^ The curing peaks obtained from DSC and FSC were used for this analysis, and the DSC and FSC data sets were analyzed separately. For DSC and FSC, the integration limits for the curing peaks were determined after performing a data smoothing step using adjacent averaging and then calculating the derivative of the heat flow to identify the beginning and ending points of curing. The curing peak was isolated by identifying the midpoint of the slope change in the derivative signal at the two bounding temperature regions. An example of this analysis method is shown in Figure S2.

Linear fitting at specific conversion values across the heating rates was performed to determine kinetic information such as the rate of the cross-linking reaction and activation energy (*E*_a_) as a function of conversion. Two isoconversional methods used for the analysis in this work, which were the Friedman and Starink equations, given as eqs 2 and 3, respectively.
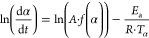
2
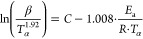
3

In these equations, α is the conversion value, *t* is time, β is the heating rate, *T*_α_ is the temperature at the respective conversion value, *A* is the pre-exponential factor, *f*(α) is the reaction model, *C* is a constant, *E*_a_ is the activation energy, and *R* the gas constant. For these two methods, the Friedman^31^ model is a differential method, and the Starink model^32^ is an integral method. Both the Friedman and the Starink methods can be considered “model-free” methods as they assume a constant reaction model throughout the full conversion process. The activation energy as a function of the extent of conversion was calculated using these two models, and results obtained for DSC experiments and FSC experiments were compared; the limits of conversion to perform these calculations were between α = 0.05 and α = 0.95.

## 3 Results and Discussion

### 3.1 Powder Characterization

**Figure 1:**
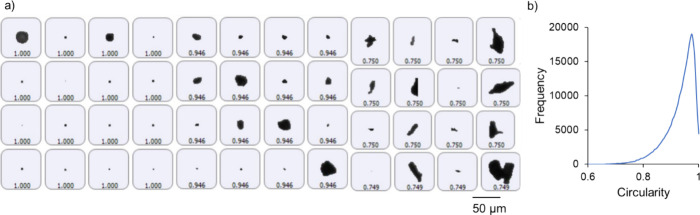
Particle shape and size distribution: (a) Particle images illustrating the varying sizes of the particles and their measured circularity values and (b) the distribution of the circularity values obtained from the characterization.

Powder feedstocks used with PBF-LB are generally spherical to ease particle deposition and packing in the powder bed,^10^ so characterizing the powder particle size and morphology was relevant to the processing even if it was not a primary concern for the calorimetry experiments. As mentioned above, only a limited number of commercially available thermoset feedstocks for PBF-LB are available, so a commercial powder coating material was sourced as the model system in this work. Figure 1 shows the results of the powder particle characterization performed on the uncured starting powder. The particle size distribution of the powder coating material was obtained based on measurements with static image analysis. The values of D_10_, D_50_, and D_90_ were 16, 47, and 90 μm, respectively. The average particle diameter, obtained as D[4,3], was 51 μm, and this value was within the ideal range of 45–90 mm for polymer PBF-LB.^10,33^ Beyond the particle size distribution, aspects of the particle shape were captured in the characterization, which would also affect processing with PBF-LB. As shown in the example images in Figure 1a, the particle population contained a broad range of particle shapes. While some images showed near circular shapes in two dimensions, other images showed irregular shapes with jagged edges. The distribution of shapes present in the population of particles was obtained based on a shape factor termed circularity. The circularity was acquired from the two-dimensional projection of the individual particle images. If the particle had a circular projection, then the circularity would have a value of 1. All other shapes would have values less than 1, decreasing more as the shape became less circular. The distribution in circularity values for the powder particles is given in Figure 1b and had a mean value of 0.93. This average circularity value, based on the work done by Grace and Ebneyamini, would generally correspond to low aspect ratio cylinders or spheroids.^34^ Aside from characterization of particle shape and size, TGA was used to observe the thermal degradation behavior of the material. As shown in Figure S3, degradation occurred in a single step, with 5% weight loss observed at approximately 350 °C. The maximum temperature used in the calorimetry experiments was 300 °C, below this threshold and before major degradation of the material at higher temperatures.

### 3.2 Curing Behavior in DSC

**Figure 2:**
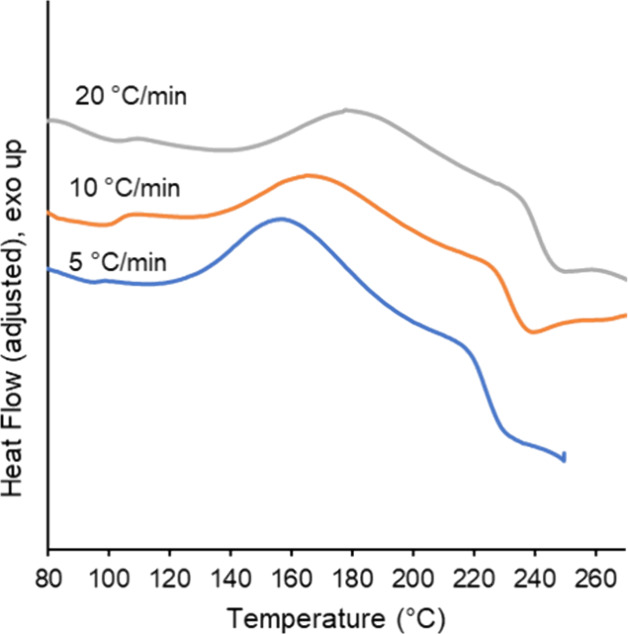
Representative DSC thermograms of the first heating cycle at each heating rate. Data collected at 10 °C/min are reprinted from Additive Manufacturing, 72, C.A. Chatham and A.L. Washington II, A framework for forming thermoset polymer networks during laser powder bed fusion additive manufacturing, 103620, Copyright (2023), with permission from Elsevier.

Understanding and modeling the curing evolution of this material required observing its exothermic signal at various heating rates. DSC is a standard technique for observing curing in thermosetting resins and was used to obtain the nonisothermal curing behavior in this work. Figure 2 shows representative thermograms of the curing during the first heating cycle for the powder coating material. Following a slight endothermic step which may be associated with water loss from the uncured powder, the exotherm associated with curing occurred over a broad temperature range and did not have a common peak shape. Instead of a single peak, the curing exotherm adopted a saddle-like shape. This behavior was associated with the curing agent used, HAA. The curing reaction between HAA and polyester occurred by esterification and produced water.^35,36^ The evolved water caused an overlapping endothermic response, affecting the heat flow curve shape measured by DSC. In other published work with polyesters cured with HAA, the endothermic signal associated with water loss could overwhelm an exothermic curing reaction, leading to a net endothermic response associated with curing.^35^ However, in this material, an exothermic signal was obtained and attributed to the curing, though the overlapping effects of curing and evolved water on the heat flow signal cannot be completely discounted and introduce a degree of uncertainty into the kinetic analysis.

Since multiple experiments were performed at each heating rate, the heat flow and enthalpy related to the curing were averaged across the samples at each heating rate for further analysis and model fitting. The total enthalpy associated with the curing and the peak temperatures are shown in Table 2. The peak temperature was defined as the temperature associated with the highest exothermic heat flow value. Comparing the heating rates, the average enthalpy value was increased for the data obtained at 10 and 20 °C/min, and the uncertainty value was reduced, suggesting that the heat flow signal was not as affected by water loss at higher heating rates. Additionally, the peak temperature increased as the heating rate increased. This change corresponded to the temperature bounds associated with curing shifting to higher temperatures, as would be expected.

**Table 2:** Enthalpy Values and Peak Temperatures Associated with the Curing Region in DSC Experiments

heating rate (°C/min)	enthalpy (J/g)	peak temperature (°C)
5	17.9 ± 9	158 ± 2
10	24.1 ± 2	174 ± 1
20	25.1 ± 0	187 ± 0

**Figure 3:**
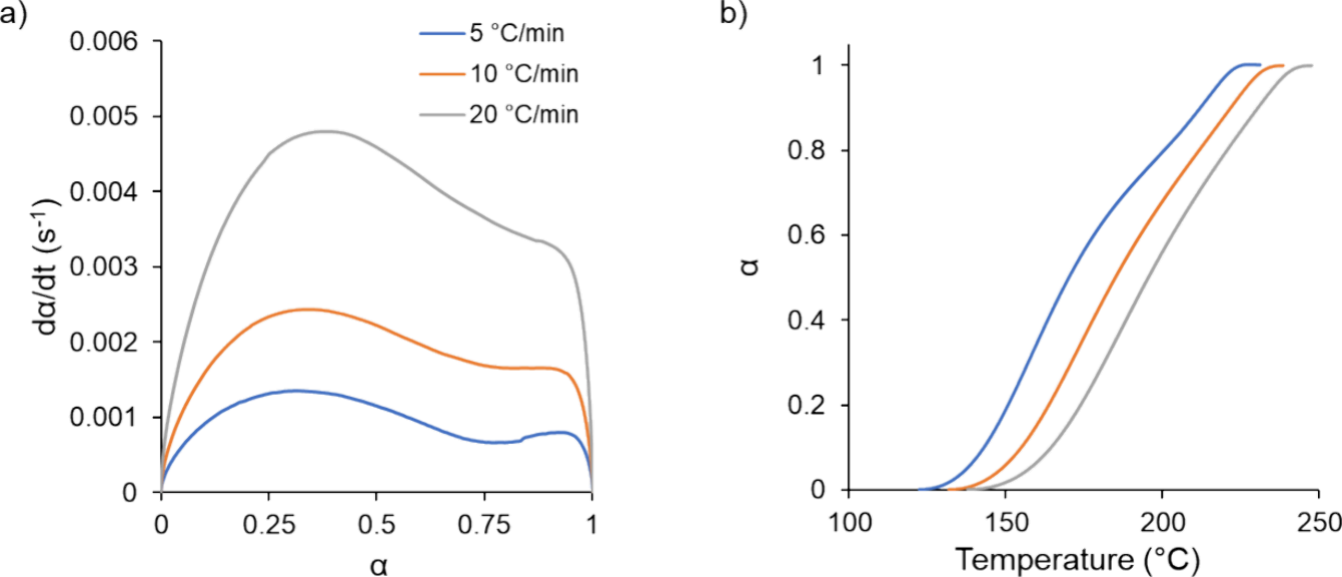
Rate of conversion (a) and conversion as a function of time (b) for the DSC experiments. Similar conversion trends were observed for all of the heating rates used in the DSC experiments.

Figure 3a shows the conversion and conversion rate data obtained from DSC. Considering the conversion rate data shown, the cure shapes for the different heating rates were similar, suggesting that the heating rate was not significantly impacting the cross-linking reaction. This result was consistent with similar studies.^7,37^ The dip in the conversion rate between roughly 30–90% conversion corresponded to the production of water during the curing. Additionally, for the conversion data shown in Figure 3b, the conversion curves did not overlap and were shifted to higher temperatures as the heating rate increased. This result further supported the suggestion that the curing behavior and mechanism were not significantly impacted at the selected heating rates, rather the temperature bounds of the curing were simply translated to higher temperatures as the heating rate increased.

**Figure 4:**
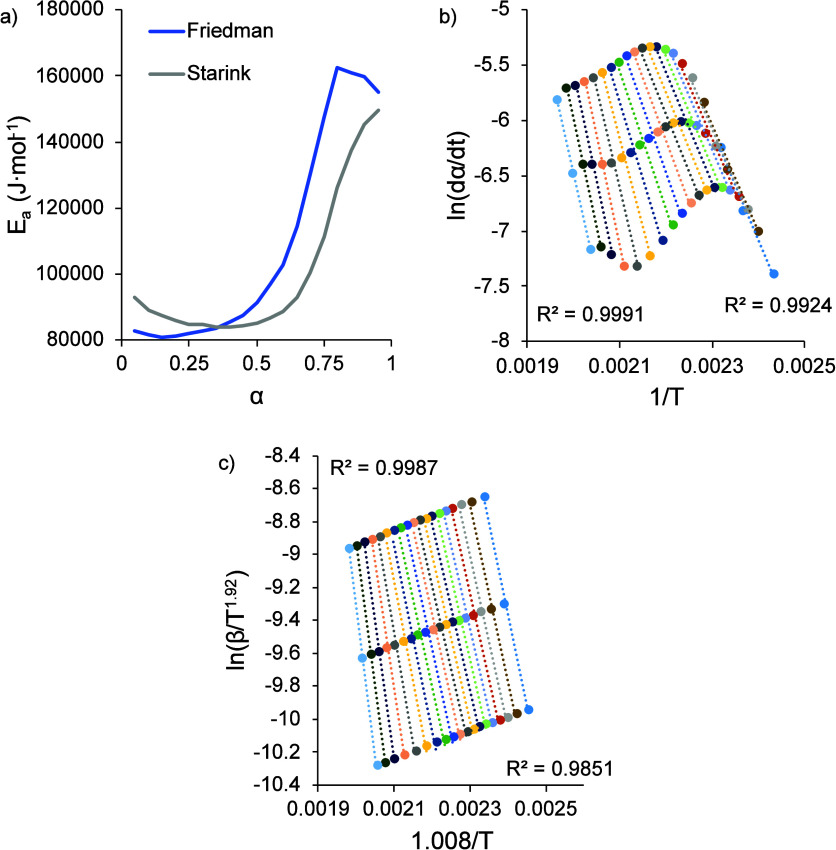
Model fitting results and data. (a) Activation energy as a function of conversion and fitting data at different conversion levels using the (b) Friedman and (c) Starink methods. The *R*^2^ values are shown for the initial and final conversion (α = 0.05 and α = 0.95, respectively).

Figure 4 shows the activation energy values and fitting data resulting from applying the two isoconversional models to the curing data. Although the two models used included one differential method (Friedman) and one integral method (Starink), the curve shapes shown in Figure 4a showed similar overall trends.^7,19^ Additionally, the activation energy was found to vary with conversion, which could suggest changes to the curing mechanism as conversion proceeds. For the two models, the activation energy decreased slightly and then did not change substantially until higher conversion values were reached, showing substantial increases at conversion values of approximately 0.5 and 0.6 for the Friedman and Starink models, respectively. For thermosets generally, the initial decrease in *E*_α_ has been suggested to result from a decreased melt viscosity as temperature increases.^38^ In previous work with polyester resins cured with HAA, the water produced during cure was suggested to act as a plasticizer, decreasing viscosity which could reduce barriers to curing after the initial curing and affect the activation energy values.^38^ Either mechanism or a combination of these mechanisms may have been present in this system. At higher conversion values, molecular chain growth began to limit mobility of the reactants, and as a result, the E_α_ value increased. shifting the curing to being diffusion controlled and consequently kinetically limited.^7,19^

Figure 4b,c show the specific terms used to perform a linear fit that determines the slope. These slopes were then used to obtain the activation energy at discrete conversion values for each isoconversional method. In these plots, each dotted line connects equivalent conversion values at the different heating rates, with the highest conversion values shown on the left most side of the plot. In general, the *R*^2^ values for the dotted lines were close to 1, indicating that the activation energy values predicted by the model were a good fit to the experimental data.

### 3.3 Curing Behavior in FSC

**Figure 5:**
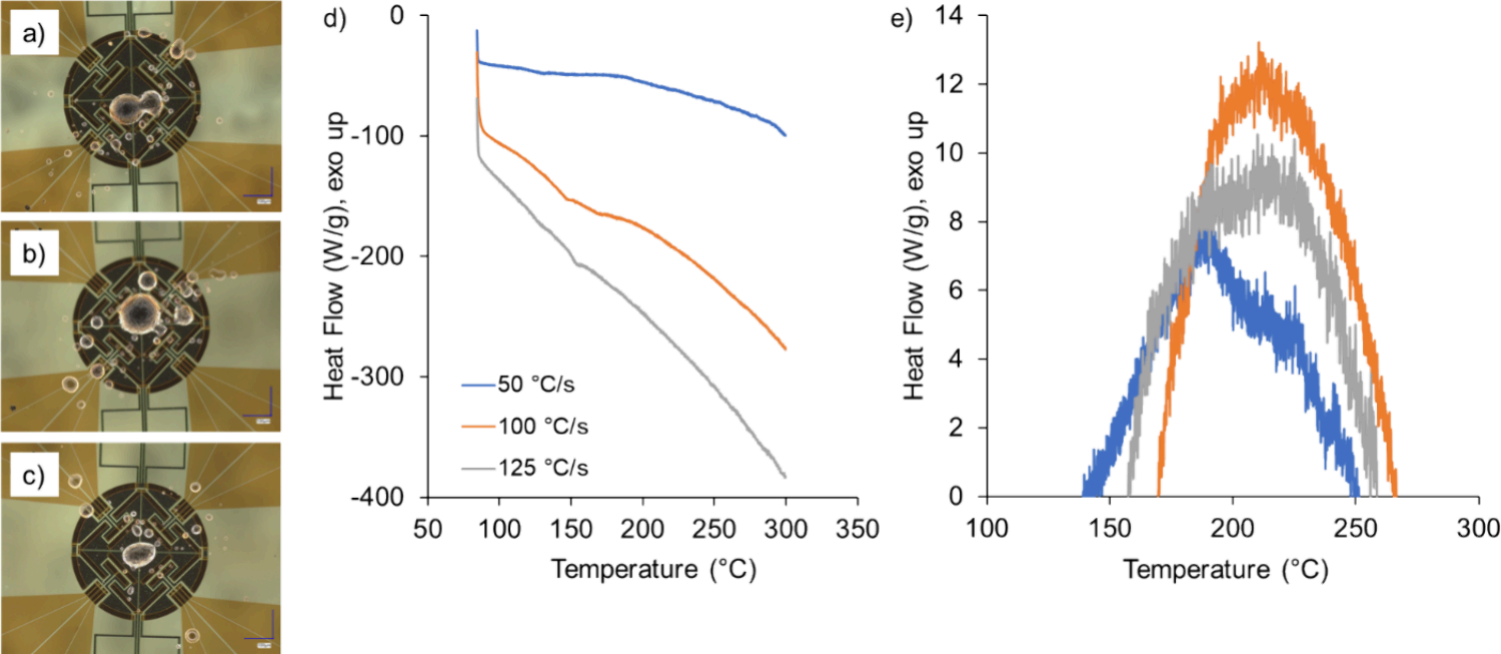
Images of the sample chip membranes used for the experiments at (a) 50, (b) 100, and (c) 125 °C/s, (d) thermograms for experiments at those heating rates, and (e) the isolated curing peaks from the FSC heat flow signal.

To compare the nonisothermal curing response at higher heating rates, similar experiments were conducted with FSC. Figure 5 shows the images of cured powder particles on the chip sensors as well as the thermograms for each of the heating rates tested. As shown in Figure 5a–c, the material placement on the chip sensors was largely in the inner diamond with some material outside this area due to difficulties with detaching individual particles from the eyelash tool. The heat flow signals obtained from FSC for these samples are shown in Figure 5d. Overall, the curing peaks were less pronounced than the curing peaks from DSC. The method used to isolate the curing peak resulted in an exothermic signal between temperatures starting at 139 to 169 °C and ending at 251 to 270 °C. While this temperature range was slightly larger than what was obtained with DSC, the observation of a broad curing peak was consistent with the DSC results. The isolated curing peaks are shown in Figure 5e. The shape of the curing peak obtained in these experiments was different than the shape observed with DSC experiments. Specifically, a single peak was observed in FSC experiments, as opposed to the saddle-shaped peak observed in DSC experiments. The change in peak shape suggested that water formation was suppressed, or its effects reduced at these heating rates since an endothermic dip was not seen in the curing peak.

Measurements of the peak temperature, enthalpy, percent conversion, *T*_g_ from the heating cycle after the nonisothermal curing step, and *T*_g_ from the heating cycle following the isothermal curing step are listed in Table 3. Compared to the results from DSC, the peak temperatures were higher, and the total enthalpy values were lower, indicating that the curing was not progressing to completion in the FSC experiments. This result was logical since the time available for curing was much shorter at the higher heating rates used in the FSC experiments. To determine the conversion value achieved at these rates, the enthalpy of the curing peak was normalized by the highest average enthalpy value measured from the DSC experiments (25.1 J/g obtained at a heating rate of 20 °C/min). The resulting conversion values were 28–34%, suggesting that the heating rates in PBF-LB may not be sufficient to fully cure the material in a single pass. Incomplete curing was also observed by analysis of *T*_g_ values from subsequent heating steps. Following the nonisothermal curing step, the values of *T*_g_ were between 75 and 78 °C. After an isothermal curing step to complete the curing, the value of *T*_g_ increased to values between 81 and 84 °C, again suggesting that full curing was not reached during the nonisothermal curing step since a higher value of *T*_g_ was attained after isothermal curing.

**Table 3:** Thermal Transition Data Obtained from FSC Measurements

heating rate (°C/s)	peak temperature (°C)	enthalpy (J/g)	conversion (%)	*T*_g_ after nonisothermal step (°C)	*T*_g_ after isothermal step (°C)
50	189	8.2	34	78	81
100	211	7.5	31	77	84
125	210	6.7	28	75	81

Although complete curing was not reached at the faster heating rates used in FSC, this result was not necessarily undesirable for PBF-LB processing. Chatham and Washington^7^ noted the need for cross-linking to occur only partially when scanning a single layer, so that powder particles in adjacent layers would be able to coalesce and fuse through further cross-linking as part printing continues. Also, incomplete curing during PBF-LB processing was experimentally observed with other thermosets, so this result was typical among published studies.^15−17^ Given differences in cross-linking reactions and mechanisms within thermosets, it is likely that there is not a single ideal level of curing to be attained during PBF-LB processing, so examining this aspect for feedstocks of interest would be an area of further study as material development continues.

**Figure 6:**
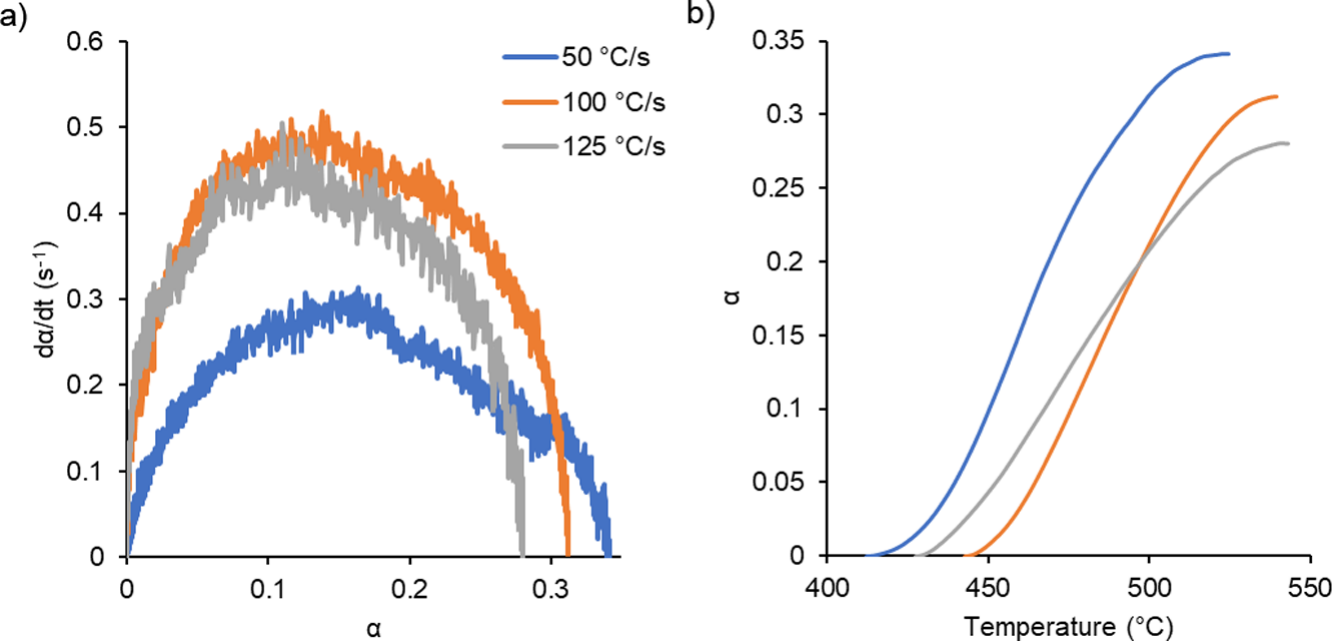
Rate of conversion (a) and conversion as a function of time (b) for the FSC experiments.

The FSC conversion data were analyzed in the same way as the DSC data and the resulting conversion and conversion rate data are shown in Figure 6. Overall, the conversion rate data had more noise associated with them, which was attributed to the low signal-to-noise ratio in the heat flow signal from the FSC experiments. The shape of the conversion rate curves showed some variation with heating rate as shown in Figure 6a. The conversion rate curve shape obtained at 50 °C/s resembled the DSC data most closely, showing a dip in the conversion rate and then a slight increase toward the end of the curing. The other two curves showed less of a dip after the highest conversion rate. Like the DSC data, the curves showed the highest conversion rate at low levels of conversion, approximately α = 0.16 at 50 °C/s, α = 0.14 at 100 °C/s, and α = 0.11 at 125 °C/s. A number of factors could be contributing to the shape changes seen in these curves. First, the degree of conversion achieved in these experiments is lower than those achieved in DSC, so these curves do not represent the complete curing process. Second, these experiments were conducted on open sensor chips in a flowing gas environment instead of sealed pans, meaning that water evolved during the curing could be transported away from the sample more quickly, possibly reducing its endothermic contribution to the heat flow signal, and third, the heating rates used were much faster allowing less time for the curing reaction to occur, possibly changing the curing kinetics. However, these factors also allowed FSC experiments to capture features of the PBF-LB process since the process is conducted in an open bed and at fast heating rates. However, a fourth factor that would not accurately represent PBF-LB processing was that limited particle coalescence was occurring in FSC experiments relative to the DSC experiments. Overall, larger amounts of particle surface area may be available during curing in FSC, which could also affect the curing process. The conversion data shown in Figure 6b also showed some differences when compared to the DSC data. The values obtained at 125 °C/s crossed over the data set for 100 °C/s as opposed to being shifted to higher temperatures. This overlap resulted from the assignment of bounding temperatures for the curing reaction. The starting and ending temperatures were less than those for the 100 °C/s data set. The result was not expected and might be associated with the sample preparation or experimental error; however, the lower degree of conversion attained at the highest heating rate was expected.

**Figure 7:**
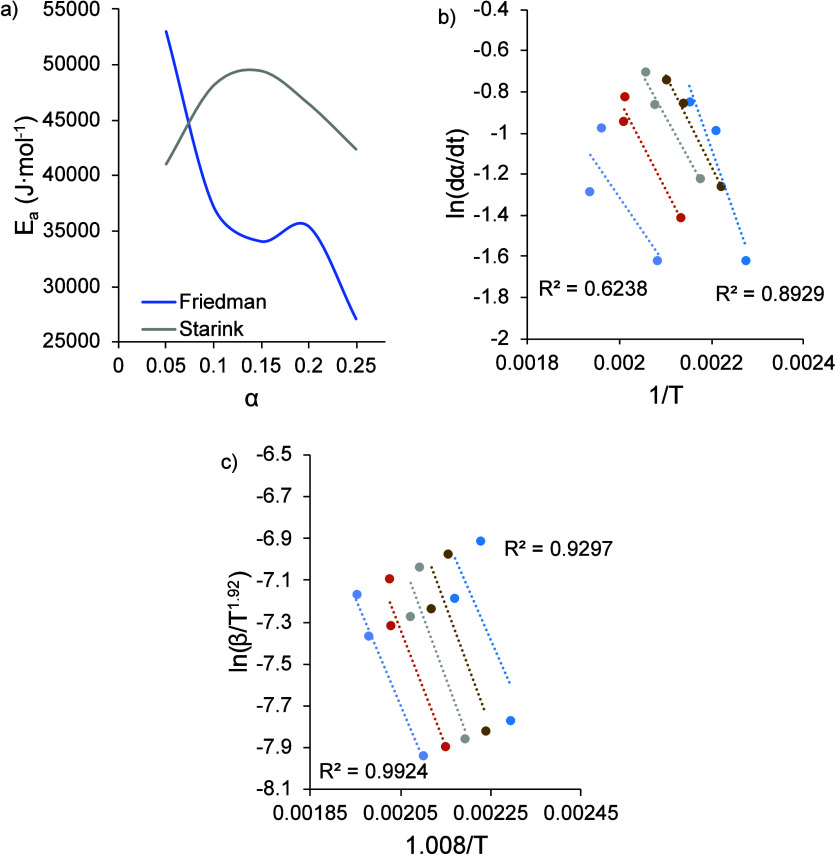
(a) Activation energy as a function of conversion for the TGIC-free polyester powder. Model fitting data at different conversion levels using the (b) Friedman and (c) Starink method. The *R*^2^ values show for the initial and final conversion (α = 0.05 and α = 0.25, respectively).

Figure 7a shows the activation energy values resulting from fitting the conversion data to the Friedman and Starink models and the model fitting data. These data were fit over a conversion range of α = 0.05 to α = 0.25, corresponding to the highest level of conversion attained at the highest heating rate in the FSC experiments. Unlike the DSC results, the shape of the activation energy curve differed for the two methods used. The Friedman method predicted a general decrease in *E*_a_ values over the conversion range, while the curve resulting from Starink method had a slight peak. The maximum activation energy was observed at 5% conversion for the Friedman model and 15% conversion for the Starink model. The value of 15% conversion corresponded approximately with the maximum conversion rate shown in Figure 6a. The reduced activation energy values seen here further supported the assertion that evolved water from esterification had a diminished effect on the curing in FSC experiments and/or that the lesser degree of particle coalescence occurring on the FSC chip sensor was leading to lower barriers to curing. Figure 7b,c shows the linear fits used to obtain the activation energy values for each isoconversional method. In comparison to the DSC results, the quality of fit for both of the models used was lower considering the *R*^2^ values. The Starink model had a higher quality fit than the Friedman model, and the fit improved at higher degrees of conversion for the Starink model. Comparing the activation energy trends obtained from the DSC and FSC experiments revealed clear differences in the curing behavior of this material. The initial activation energy, representing the energy threshold required to initialize curing, was higher for nonisothermal curing at the heating rates used in DSC. Additionally, activation energy values for DSC were larger than FSC activation energy values at all conversion values; considering the heating rates and the amount of cross-linking achieved in FSC experiments, even lower amounts of cross-linking would be expected to occur at the higher heating rates in PBF-LB. Consequently, the demonstrated lowering of the extent of cross-linking would coincide with reduced evolved water.

### 3.4 Role of DSC and FSC Experiments in Assessing Curing Behavior during PBF-LB Processing

Calorimetry experiments are widely used and provide valuable information about the thermal transitions and structure of polymers. They have been applied to understand the effects of processing on polymers, including processing with additive manufacturing.^39−43^ Simulating a processing environment is more challenging with these experiments since aspects like heating/cooling rates, sample sizes, environmental conditions, and the presence or absence of shear flows may be different for calorimetry experiments as compared to actual processing operations. However, these experiments have a role to play in designing and understanding processing operations.

Beyond analysis of polymers generally with calorimetry, methods for evaluating curing with DSC are well-established, and information about the curing process/mechanism can be extracted from these results. With this baseline, fundamental information can be used to screen thermoset materials for compatibility with PBF-LB through measuring heat evolution during curing, establishing isothermal and nonisothermal curing kinetics, and calculating activation energy trends. Including FSC as a secondary screening experiment, introduces an ability to capture the curing behavior at higher heating rates and allows for an understanding of the expected conversion levels with PBF-LB. Changing the heating rates also allows for a better understanding of how the chemical reaction associated with curing could be affected by PBF-LB processing. While the polyester powder used in this work may not be the best candidate for actual printing in PBF-LB, it provided an example of how evolved water removal during curing may affect the curing process and how this change may affect isoconversional analysis.

This work explored a relatively simple thermal program and demonstrated some similarities with larger scale printing studies. Chatham and Washington used an isoconversional analysis method to predict the extent of cure of the same polyester powder used in this work, where the percent conversion only reached roughly 29% of full cure when simulating heating of the laser on a single layer of material. This value was within range of the experimentally measured percent conversion seen in FSC characterization, reflected in Table 3, suggesting a good correlation between experimental and computational results. Beyond the protocols used in this work additional features of PBF-LB processing such as repeated heating and cooling can be incorporated into experimental protocols to gain additional insight into the compatibility of the material and the processing method. Ultimately, this work provided an initial demonstration of how DSC and FSC can be paired to screen materials and reduce the number of printing trials needed to evaluate feedstock materials for PBF-LB.

## 4 Conclusions

In this work, complementary curing studies were conducted using two types of calorimetry methods, DSC and FSC, with a motivation of using these results to better understand curing of thermoset feedstocks during PBF-LB processing. The results of these experiments and the corresponding isoconversional analysis showed that the curing process was altered when heating rates were increased. Specifically, these experiments indicated that conversion values on the order of 30% were achieved with FSC, while full curing was achieved with DSC. These conversion values from FSC experiments were similar to previously published results obtained through applying isoconversional models to thermal profiles from PBF-LB, supporting the use of FSC as a reasonable proxy for PBF-LB printing studies. Additionally, the FSC results were consistent with the results of published PBF-LB studies with other thermoset feedstocks in that complete conversion was not achieved in PBF-LB processing.

When combined, the results from DSC and FSC provided two perspectives on the curing of the material that could inform the use of thermosets with PBF-LB. Beyond differences in the conversion achieved at faster heating rates, differences in the experimental setup for DSC and FSC also influenced the results. In FSC experiments, water produced during esterification did not hinder the curing reaction to the same extent as it did in DSC experiments, which may more closely mimic the processing conditions in PBF-LB. This difference was at least one factor that led to lower activation energy values for curing at higher heating rates. The general applicability of this trend could be assessed in future studies with other thermosetting resins that cure by reactions other than esterification and by designing more complicated thermal programs that would simulate other aspects of PBF-LB processing such as the curing of adjacent layers with repeated laser passes. Overall, the results of this work showed that combining DSC and FSC experiments as tiered screening tools would be useful for developing thermosets for PBF-LB and evaluating the types of curing reactions that would be most compatible with PBF-LB processing.

## Supporting Information Available

Example thermogram from DSC, illustration of method for isolating the curing peak from FSC, characterization of thermoset polymer with TGA, and tabulated sample data (temperatures and mass values) from DSC and FSC experiments (PDF)
