# Supplementary material for: Investigating Fast Scanning Calorimetry and Differential Scanning Calorimetry as Screening Tools for Thermoset Polymer Material Compatibility with Laser-Based Powder Bed Fusion
Source: ACS Appl Polym Mater. 2025 Jan 13;7(2):719–28. doi: 10.1021/acsapm.4c03052 (PMC11773405; doi:10.1021/acsapm.4c03052)
Supplement: Supplementary file 1 — ap4c03052_si_001.pdf [file ap4c03052_si_001.pdf]

Supporting Information

Investigating Fast Scanning Calorimetry and Differential Scanning Calorimetry as Screening Tools for Thermoset Polymer Material Compatibility with Laser-Based Powder Bed Fusion

Malik A. Blackman[a], Meisha L. Shofner[a]*, and Camden A. Chatham[b]*

[a] School of Materials Science and Engineering, Georgia Institute of Technology, Atlanta, Georgia 30332, USA

[b] Advanced Engineering Division, Savannah River National Laboratory, Savannah River Site, Aiken, South Carolina 29808, USA

*Email: meisha.shofner@mse.gatech.edu; camden02.chatham@srnl.doe.gov

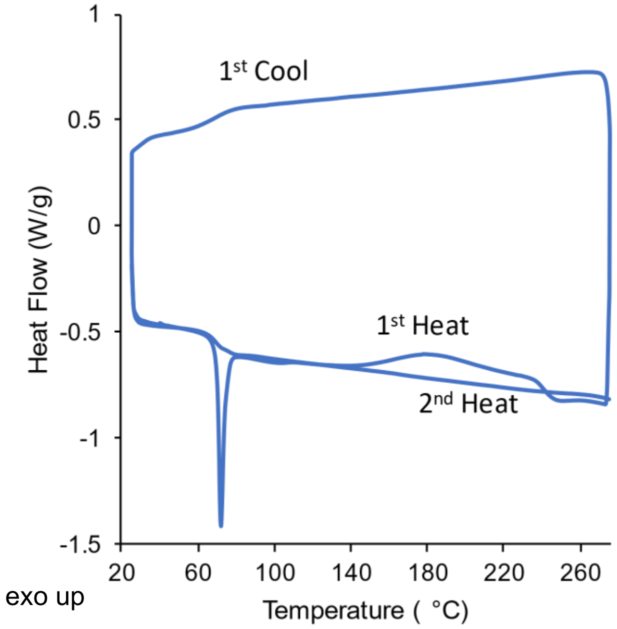

Figure S1. Heat-cool-heat example of polyester powder. The first heat illustrates the curing peak seen from heating at 20 °C/min, while the second heat cycle does not show an exothermic reaction, indicating that curing has completed in the first heat.

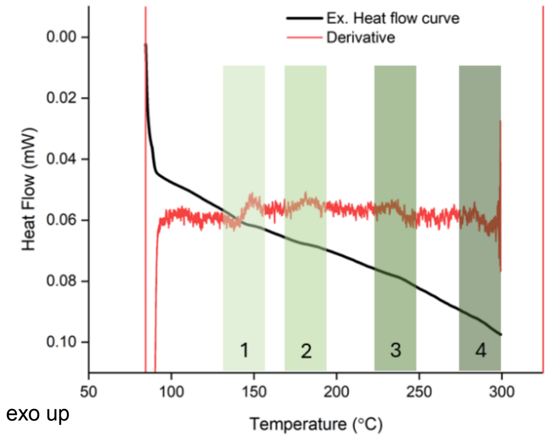

Figure S2. Example heat flow curve that has been smoothed and its derivative taken to identify key thermal regions in the data. Region 1 was identified as an endothermic transition, region 2 was the onset of cure, the end of cure was seen in region 3, and region 4 was continued heating of the thermoset.

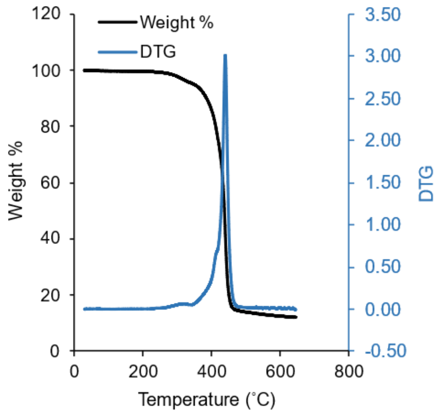

Figure S3. Percent weight loss as a function of temperature for the TDA 61 polyester powder. At 350 °C, the material experienced a 5% weight loss, which led to the end temperature for testing to be at 300 °C for all samples.

Table S1. Temperature range and mass values for DSC samples.

Sample	Start Temperature (˚C)	End Temperature (˚C)	Mass (mg)
5 °C/min	0	250	9.13 ± 0.54
10 °C/min	25	275	7.40 ± 1.08
20 °C/min	25	275	7.33 ± 1.52

Table S2. Mass values for FSC samples.

Sample	Mass (ng)
50 °C/s	533
100 °C/s	634
125 °C/s	372
